# Developing an antibiogram for empiric antibiotic prescribing for adult non-spinal orthopaedic infections in a developing world setting

**DOI:** 10.1007/s00590-023-03718-4

**Published:** 2023-09-15

**Authors:** Ashley Arakkal, Chad M. Centner, Thomas Hilton, Marc Nortje, Michael Held, Stephen Roche, Adrian J. Brink, Marc Mendelson, Maritz Laubscher

**Affiliations:** 1 Orthopaedic Research Unit, Division of Orthopaedic Surgery, H49 OMB, Groote Schuur Hospital, University of Cape Town, Cape Town, 7925 South Africa; 2 Division of Medical Microbiology, University of Cape Town and National Health Laboratory Service, Groote Schuur Hospital, Cape Town, South Africa; 3 Division of Infectious Diseases and HIV Medicine, Department of Medicine, Groote Schuur Hospital University of Cape Town, Cape Town, South Africa

**Keywords:** Fracture, Infection, PJI, FRI

## Abstract

**Purpose:**

Empiric antibiotic strategies in the treatment of fracture-related infections, chronic osteomyelitis, prosthetic joint infection, and septic arthritis should be based on local microbiological antibiograms. This study aims to describe the microbiology and review the antibiogram profiles of bacterial isolates from patients undergoing surgical treatment for non-spinal orthopaedic infections, to identify the most appropriate empiric antibiotic strategy.

**Methods:**

A retrospective review was performed of all cases of non-spinal orthopaedic infections treated surgically from 1 January 2018 to 31 December 2018. The National Health Laboratory Service microbiology database was used to identify all intra-operative microbiological specimens obtained from orthopaedic patients, and data were correlated with the orthopaedic surgical database. Cases were divided into fracture-related infections, chronic osteomyelitis, prosthetic joint infection, and septic arthritis. Antibiotic susceptibility data were used to predict the efficacy of different empiric antibiotic regimens.

**Results:**

A total of 107 cases were included in the study; 184 organisms were cultured. Overall, the most common organism cultured was *Staphylococcus aureus* (25%) followed by *Acinetobacter baumannii* (9%), *Enterococcus faecalis* (7%) and *Enterobacter cloacae* (5%). Across all categories the oral antibiotic combination with the highest effectiveness (81%) would have been a combination of co-trimoxazole, ciprofloxacin and amoxicillin. The most effective intravenous antibiotic combination would have been either piperacillin–tazobactam, amikacin and vancomycin or meropenem and vancomycin; 90% of tested isolates were susceptible to either of these combinations.

**Conclusion:**

Antibiogram profiles can serve to guide to empiric antibiotic choice in the management of different categories of non-spinal orthopaedic infections.

## Introduction

Common orthopaedic infections including septic arthritis, acute osteitis, chronic osteomyelitis (COM), fracture-related infections (FRI), prosthetic joint infections (PJI), and spinal infections are often associated with significant morbidity [1–3]. The incidence of septic arthritis ranges from 2 to 29 per 100,000, increasing in populations with lower socioeconomic status [4–6]. The incidence of chronic osteomyelitis has been described as up to 21.8 per 100,000 person/years, and the incidence of PJI ranges from 1 to 2% in postoperative patients [7–9]. FRI occur in 2% to 30% of cases postfracture fixation, depending on the fracture type and whether it was an open or closed fracture [10, 11]. Orthopaedic infections also have a significant economic burden with the costs of the management of patients with infected fracture fixation and PJI increasing 2–6 times compared to uninfected cases [12–14]. Management usually includes debridement of devitalised tissue followed by microbiological sampling, dead space management that may include local antibiotics, and systemic antibiotic therapy. Empiric systemic antibiotics are usually initiated prior to microbial culture-directed treatment [3, 15]. The use of empiric antibiotics in orthopaedic infections leads to substantially higher cure rates compared to delayed antibiotic treatment, started once antibiotic sensitivities are known [16]. Empiric antibiotic selection should be based on local prevalence of bacteria and their antibiotic resistance patterns [17]. However, such data are often lacking in resource-limited settings, resulting in recommendations extrapolated from international guidelines. This study was undertaken to investigate the aetiology and antibiotic sensitivity patterns of orthopaedic infections at our tertiary referral orthopaedic centre in Cape Town, South Africa, with the aim of implementing empiric antibiotic recommendations based on the antibiogram for our unit.

## Methods

A retrospective review was performed of all cases of orthopaedic infections treated surgically from 1 January 2018 to 31 December 2018 at Groote Schuur Hospital, Cape Town, South Africa. The National Health Laboratory Services (NHLS) diagnostic microbiology laboratory database was used to identify all intra-operative microbiological specimens obtained from orthopaedic patients. Patient data were correlated with the orthopaedic surgical database hosted in REDCap (REDCap 9.5.36©; 2021 Vanderbilt University) [18, 19]. Acute and chronic infections were included. Cases of spinal infections as well as specimens collected from non-orthopaedic sites were excluded. Cases were divided into the relevant infection categories, namely septic arthritis, COM, FRI and PJI. Where the same organism was cultured from multiple specimens taken from the same patient, the bacterium was counted only once when calculating number of bacteria, antibiograms and antibiotic coverage if the antibiotic susceptibility findings were the same.

Chronic osteomyelitis was defined as at least 6 months of symptoms of infection with one or more of the following: sinus, abscess or purulence at the time of surgery; at least one bacterium cultured from the site of infection obtained following debridement; or histology suggestive of chronic osteomyelitis [15].

FRI was defined according to the criteria proposed by Metsemakers et al. consisting of one of the following occurring postsurgery for fracture fixation: fistula, sinus or wound breakdown (that communicates to the bone or implant); purulent drainage or the presence of pus during surgery or presence of microorganisms in deep tissue specimens confirmed by histopathological examination [1].

PJI was defined using the international consensus group definition [20] as patients who met one major criterion—two positive periprosthetic cultures with phenotypically identical bacteria or a sinus tract communicating with the joint—or ≥ 3 minor criteria from:

- Elevated serum C-reactive protein and/or erythrocyte sedimentation rate,
- Elevated synovial fluid white blood cell count,
- Elevated synovial fluid polymorphonuclear neutrophil percentage,
- Positive histological analysis of periprosthetic tissue or
- A single positive culture from intra-operative specimens

At each operation, deep samples of infected tissue and/or fluid were collected and submitted for bacterial culture. A structured, sterile sampling technique was used, where each individual specimen is taken with clean sterile instruments after thorough debridement according to the policy of the unit. We take a minimum of 4 samples as standard.

Tissue samples were processed by manual crushing in saline and then inoculated onto 4% horse blood agar, brucella agar, brucella agar supplemented with nalidixic acid, MacConkey agar and boiled blood agar. Blood agar and Brucella plates were incubated anaerobically at 37 °C, MacConkey agar plates, aerobically and boiled blood agar in 5% CO2 atmosphere. In addition, liquid culture was performed in Robertson’s cooked-meat broth. Cultures were examined daily for 5 days and up to 14 days for suspected prosthetic joint infection. Up to 3 organisms were purified from a mixed growth; however, any growth of *S. aureus*, beta-haemolytic streptococci or *P. aeruginosa* was selected from mixed samples. Identification was performed using Vitek 2 (BioMérieux, Marcy-l'*Étoile*, France), except for beta-haemolytic streptococci which were grouped by Lancefield antigen agglutination. Anaerobic organisms were not routinely speciated in the laboratory. Susceptibility testing was performed by Vitek 2, disc diffusion or gradient diffusion testing using Etest (BioMérieux) as appropriate, except for beta-haemolytic streptococci, which were assumed to be susceptible. Subsequently, aggregated antibiograms were generated: after deduplication by patient and organism, the sensitivity of each antibiotic combination to the various categories of infection was evaluated.

Ethics approval (UCT HREC reference 559/2020) as well as institutional permission was obtained prior to data collection. Statistical analysis was performed using Stata 14.2 (StataCorp LP, College Station, TX).

## Results

### Patient demographics and infection types

**Fig. 1:**
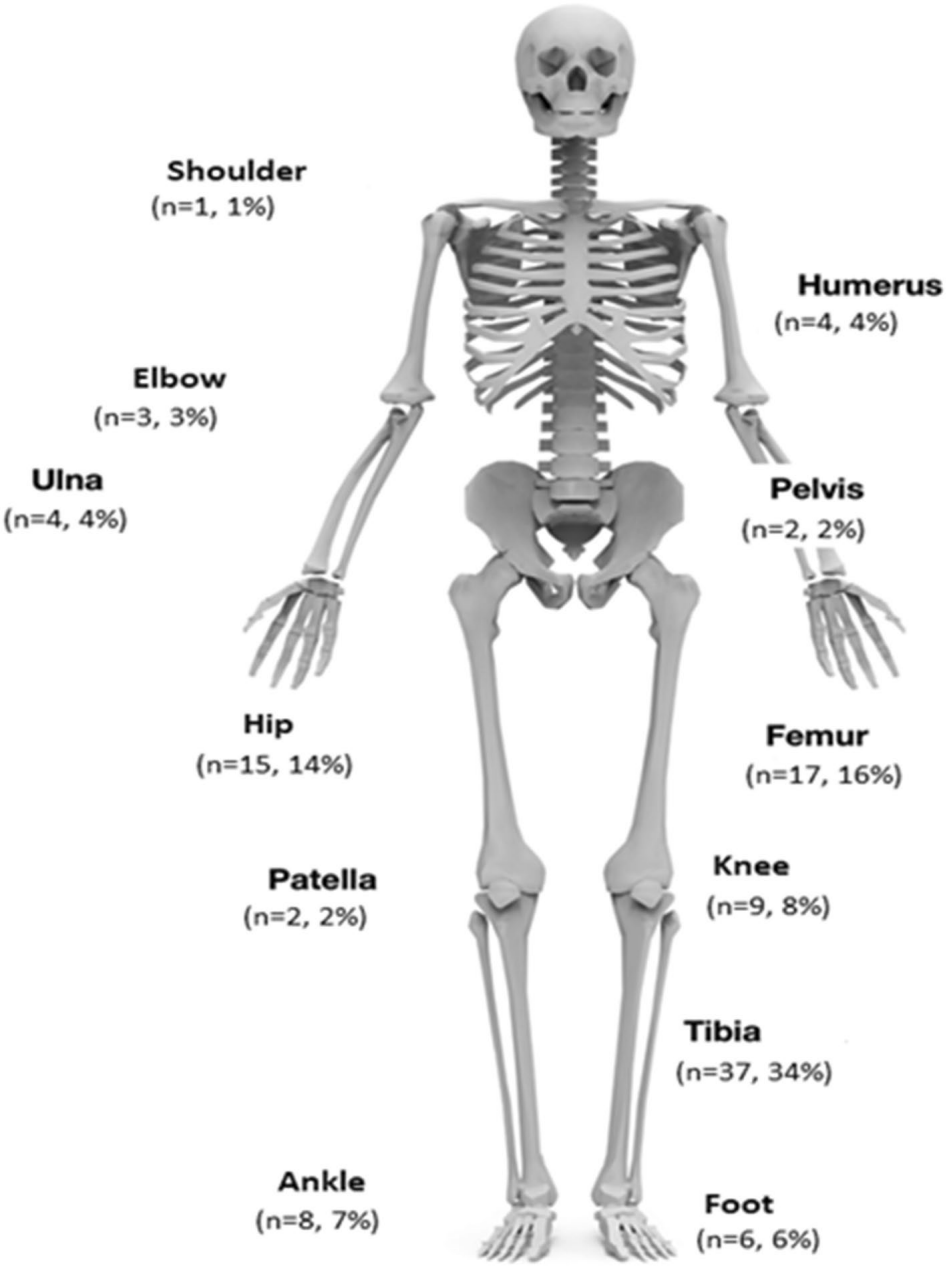
Number of patients per site of infection [15]

A total of 107 cases were included, seventy-six (71%) of whom were males. The median age was 37 years with an interquartile range of 19. Thirty-seven of the patients had more than 1 organism cultured. FRI was the leading cause of non-spinal infection in our unit, followed by COM, PJI and lastly septic arthritis (Table 1). The number of patients with infection at different anatomical sites is illustrated in Fig. 1, with tibia, hip and knee infections predominating.

**Table 1:** Patients by category of infection

Category of infection	*N* (% of total)	Sex (M/F)
Total	107 (100)	76/31
Fracture-related infection	48 (45)	37/11
Chronic osteomyelitis	36 (34)	28/8
Prosthetic joint infection	15 (14)	6/9
Septic arthritis	8 (7)	5/3

### Microbiology

Overall, a total of 184 bacteria were cultured from the 107 cases (Table 2). Infections caused by Gram-positive bacteria were twice as common as Gram-negatives, with *Staphylococcus aureus* being the predominant bacterium isolated. In terms of frequency of bacteria causing different types of orthopaedic infection in our unit*. S. aureus* and Gram-negative bacteria in the Enterobacterales order (*Escherichia coli, Klebsiella pneumoniae,* etc.) were the most common in the FRI, PJI and COM categories causing approximately 25 percent of infections each, with other bacteria having lower contributions. In the SA category the most common bacteria were *S. aureus* and coagulase-negative staphylococci.

**Table 2:** Organisms cultured by frequency as well as number of patients with those organisms

	Bacteria (*n* = 184)		Patients (*n* = 107)	
	*n*	%	*n*	%
Staphylococci				
*Staphylococcus aureus*	46	25	46	43
*Coagulase-negative staphylococci*	17	9	17	16
*Staphylococcus epidermidis*	6	3	6	6
*Staphylococcus hominis*	4	2	4	4
*Staphylococcus lugdunensis*	2	1	2	2
*Staphylococcus capitis*	1	1	1	1
*Staphylococcus pseudintermedius*	1	1	1	1
*Staphylococcus simulans*	1	1	1	1
*Staphylococcus xylosus*	1	1	1	1
*Other coagulase-negative staphylococci*	1	1	1	1
Enterobacterales				
*Enterobacter cloacae complex*	13	7	13	12
*Proteus mirabilis*	8	4	8	7
*Klebsiella pneumoniae*	5	3	5	5
*Serratia marcescens*	4	2	4	4
*Morganella morganii*	4	2	4	4
*Escherichia coli*	3	2	3	3
*Klebsiella oxytoca*	2	1	2	2
*Proteus vulgaris*	2	1	2	2
*Citrobacter braakii*	1	1	1	1
*Citrobacter koseri*	1	1	1	1
*Enterobacter aerogenes*	1	1	1	1
*Escherichia hermannii*	1	1	1	1
*Pantoea species*	1	1	1	1
*Providencia stuartii*	1	1	1	1
Non-fermenting gram-negative bacilli				
*Acinetobacter baumannii* complex	17	9	17	16
*Pseudomonas aeruginosa*	10	5	10	9
*Stenotrophomonas maltophilia*	1	1	1	1
*Acinetobacter Iwoffi*	1	1	1	1
*Acinetobacter ursingii*	1	1	1	1
*Pseudomonas stutzeri*	1	1	1	1
*Sphingomonas paucimobilis*	1	1	1	1
Gram-positive cocci				
*Enterococcus faecalis*	10	5	10	9
*Streptococcus group A*	9	5	9	8
*Streptococcus group G*	2	1	2	2
*Enterococcus casseliflavus*	1	1	1	1
*Micrococcus species*	1	1	1	1
*Streptococcus group B*	1	1	1	1
Gram-positive bacilli				
*Corynebacterium striatum*	6	3	6	6
*Bacillus cereus*	4	2	4	4
*Corynebacterium species*	2	1	2	2
*Actinomyces odontolyticus*	1	1	1	1
*Bacillus species*	1	1	1	1
*Corynebacterium minutissimum*	1	1	1	1
*Corynebacterium urealyticum*	1	1	1	1
Other				
*Shewanella algae*	1	1	1	1
*Gardnerella vaginalis*	1	1	1	1

In terms of major resistance patterns of importance, for Gram-positives, there were no cases of vancomycin-resistant *Enterococci* (VRE). Methicillin-resistance was found in 24% of *S. aureus*; however, sensitivity to cotrimoxazole, ciprofloxacin and vancomycin were 76%, 80% and 100%, respectively. CoNS group bacteria were fully sensitivity to vancomycin.

For Gram-negative infections, although 44% of *Klebsiella spp.* were extended-spectrum beta-lactamase (ESBL)-producers, rates of antibiotic sensitivity in Enterobacterales as a whole, varied considerably, ranging from 33% for co-amoxiclav to 100% for ertapenem. Although we did not isolate any carbapenem-resistant Enterobacterales, the proportion of carbapenem resistance in *A. baumannii* was 80%, and only 20% of *A. baumanii* isolates were sensitive to ciprofloxacin or gentamicin. Sensitivity to tigecycline was maintained at 100%. Relatively lower rates of antibiotic resistance were found to *Pseudomonas* spp*.* with 91% sensitivity to cefepime and 82% sensitivity to piperacillin–tazobactam.

We went on to determine what proportion of our empiric antibiotic choices for each group of orthopaedic infections encountered, were ‘appropriate’ in terms of delivering coverage for the bacteria causing the infection. We found concordance in terms of oral or intravenous empiric regimens across all infection types and high coverage rates from the chosen regimens (Table 3).

**Table 3:** Sensitivities of microorganisms to combinations of antibiotics by categories of infection. Isolates tested show number of specimens tested for sensitivity to the drug combination. Cover shows specimens that were sensitive to a particular antibiotic combination expressed as an absolute number and a percentage of the total number of isolates. For the Co-trimoxazole, ciprofloxacin combination number in parenthesis illustrates the cover with addition of a beta lactam to cover *Streptococci sp*. and *Enterococci sp*

Antibiotic combination	Fracture-related infections (*n* = 69)		Chronic osteomyelitis (*n* = 57)		Prosthetic joint infection (*n* = 46)		Septic arthritis (*n* = 12)		All isolates (*n* = 184)	
	Isolates tested (*n*)	Cover (*n*, %)	Isolates tested (*n*)	Cover (*n*, %)	Isolates tested (*n*)	Cover (*n*, %)	Isolates tested (*n*)	Cover (*n*, %)	Isolates susceptible of all isolates	Cover (%) out of all isolates
Cloxacillin/flucloxacillin	39	23, 33	39	32, 56	20	13, 28	9	7, 58	75/184	41
Co-amoxiclav	51	35, 41	48	35, 61	30	17, 37	10	7, 58	94/184	51
Co-amoxiclav + ciprofloxacin	67	50, 72	57	49, 86	41	25, 54	11	10, 83	134/184	73
Co-trimoxazole + ciprofloxacin (+ amoxicillin)	69	54(60), 78(87)	57	42(51), 74(89)	44	25(28), 54(61)	11	7(10), 58(83)	128(149)/184	70(81)
Ciprofloxacin + rifampicin	67	54, 78	57	47, 82	41	26, 57	10	8, 67	135/184	73
Cloxacillin + gentamicin	64	52, 75	55	50, 91	40	24, 52	11	10, 83	136/184	74
Vancomycin + gentamicin	69	61, 88	56	52, 93	45	32, 70	11	10, 83	155/184	84
Meropenem + vancomycin	69	62, 90	57	55, 96	45	38, 83	11	10, 83	165/184	90
Piperacillin–tazobactam + amikacin + vancomycin	69	61, 88	57	55, 96	45	40, 87	11	10, 83	166/184	90
Piperacillin–tazobactam + vancomycin	69	60, 87	57	54, 95	45	37, 80	11	10, 83	161/184	88

## Discussion

Fracture-related infection, chronic osteomyelitis and prosthetic joint infections are the predominant orthopaedic infections managed at our tertiary academic teaching hospital in Cape Town. Septic arthritis of native joints was relatively uncommon, which may reflect management at lower level of care hospitals. Overall, *S. aureus* was the commonest infecting bacteria, the majority of which were methicillin sensitive. Enterobacterales were the commonest cultured Gram-negative bacteria isolated.

Not all bacteria underwent susceptibility testing to every antibiotic or antibiotic combination, as inherent resistance is inferred to some antibiotics. However, comparing our bacterial profiles to other published studies, we found lower rates of *S. aureus* (26%) infection in our FRI cohort compared to studies by Hellebrekers et al. (41%) and Rupp et al. (37%), although our incidence of *A. baumannii* and *E. cloacae* was higher[16, 21, 22]. The balance of *S. aureus* and Enterobacterales in our COM infections was similar to other South African published cohorts [15, 23]. In the PJI category, the most common bacteria—Enterobacterales and *S. aureus—*differed from those published by Moran et al*.* from the UK and in the Australian study by Peel et al. where most organisms were *S. aureus* and coagulase-negative *Staphylococci* (CoNS) [3, 24]. The predominance of *S. aureus* as the cause of septic arthritis is not surprising, although our higher prevalence of CoNS infections was unexpected.

Our finding of no cases of vancomycin-resistant enterococci is in keeping with the fact that VRE is a relatively rare phenotype in South Africa [25]. In addition, our overall prevalence of methicillin-resistant *S. aureus* (24%) is similar to previous South African studies and equivalent to the UK [3, 6, 15].

We used our resistance data to calculate institution-specific empiric antibiotic choices for each category of orthopaedic infection. Overall, the combination of oral antibiotics with the best coverage was co-trimoxazole, ciprofloxacin and a beta-lactam (for Enterococcus and Streptococcus cover) with 81% of tested isolates sensitive to this combination. However, the side effects of prolonged quinolone therapy necessitate consideration when balancing risk–benefit equation to include this antibiotic in an empiric regimen. An empiric oral antibiotic regime is used in culture-negative cases where the clinical diagnosis is made by the presence of other major criteria, in the absence of an aetiological diagnosis. Culture-negative infections are reported in 7–34% of orthopaedic infections in different series [15]. One limitation of a standard oral empiric regimen being used for culture-negative infections is that fastidious bacteria that are difficult to culture may not be covered.

Our usual practice is to start intravenous empiric antibiotics immediately following surgery and tissue sampling, while awaiting culture results. In our setting, either combination therapy with piperacillin–tazobactam, amikacin plus vancomycin or meropenem plus vancomycin, would have provided antibiotic cover for cultured bacteria in 90% of cases and piperacillin–tazobactam plus vancomycin alone (thus reducing the risk of nephrotoxicity) in 88% in our total cohort.

In terms of individual types of orthopaedic infections, the most effective oral empiric antibiotic combinations for FRI, COM and PJI were co-trimoxazole, ciprofloxacin and amoxicillin, covering more than 87% of infections for FRI and COM, but only 61% for PJI. The oral combination data can help to inform antibiotic choice in culture-negative cases (where an infection was diagnosed through the presence of other criteria. Similarly, the top performing combinations for intravenous regimens, meropenem plus vancomycin and piperacillin–tazobactam, amikacin, plus vancomycin provided robust sensitivity between 83 and 90%.

Although the number of isolates in the septic arthritis category was low, the oral antibiotic combinations of ciprofloxacin plus co-amoxiclav and co-trimoxazole once again had coverage rates over 80%, as did ciprofloxacin and amoxicillin. This reflects the higher incidence of staphylococcal infections in this group. Coverage from all intravenous and oral combinations was equivalent.

Intravenous antibiotics are typically preferred to oral antibiotics for empiric treatment of postsurgical orthopaedic infections since enteral absorption may be compromised during the early postoperative period. In addition, higher doses of many antibiotics can be administered intravenously, which may be beneficial due to the initial high bacterial load at the infection site. Intravenous therapy is generally continued until culture results are available.

Limitations of the study were that it was retrospective, with generally small numbers particularly when categorising by type of infection. The microbiology represents orthopaedic infections from a single orthopaedic unit and is not necessarily representative of all orthopaedic infections across South Africa. In addition, with the study data being from specimens collected in 2018 it is possible that the antibiotic resistance patterns have changed due to increasing development of bacterial resistance over time.

## Conclusion

This study illustrates the process of developing a local antibiogram to inform institutional recommendations for the selection of empiric therapy across non-spinal orthopaedic infections. In the FRI, COM and PJI categories the most prevalent organisms were S. aureus and the Enterobacterales. In the SA category the most prevalent organisms were S. aureus and coagulase-negative staphylococci. Across all categories the oral antibiotic combination with the highest effectiveness would have been a combination of co-trimoxazole, ciprofloxacin and amoxicillin which can be used as an option in the treatment of culture-negative infections. The most effective intravenous antibiotic combination across all categories of infection would have been either piperacillin–tazobactam, amikacin and vancomycin or meropenem and vancomycin. We recommend regular surveillance to monitor changes in antibiotic resistance patterns and similar reporting of studies at other institutions to aid in understanding the differences in antibiotic resistances patterns across geographic regions and for identifying stewardship initiatives and targets for education.

## References

[1] Metsemakers WJ, Morgenstern M, McNally MA (2018). Fracture-related infection: a consensus on definition from an international expert group. Injury..

[2] Trampuz A, Zimmerli W (2006). Diagnosis and treatment of infections associated with fracture-fixation devices. Injury.

[3] Moran E, Masters S, Berendt AR, McLardy-Smith P, Byren I, Atkins BL (2007). Guiding empirical antibiotic therapy in orthopaedics: The microbiology of prosthetic joint infection managed by debridement, irrigation and prosthesis retention. J Infect.

[4] García-Arias M, Balsa A, Mola EM (2011). Septic arthritis. Best Pract Res Clin Rheumatol.

[5] Morgan DS, Fisher D, Merianos A, Currie BJ (1996). An 18 year clinical review of septic arthritis from tropical Australia. Epidemiol Infect.

[6] Ross JJ (2017). Septic arthritis of native joints. Infect Dis Clin North Am.

[7] Kremers HM, Nwojo ME, Ransom JE, Wood-Wentz CM, Joseph Melton L, Huddleston PM (2014). Trends in the epidemiology of osteomyelitis a population-based study, 1969 to 2009. J Bone Joint Surg-American.

[8] Ahmed SS, Haddad FS (2019). Prosthetic joint infection. Bone Joint Res..

[9] Earnshaw P (2001). Controversies in total knee replacement. J Bone Joint Surg Br..

[10] Foster AL, Moriarty TF, Trampuz A (2020). Fracture-related infection: current methods for prevention and treatment. Expert Rev Anti Infect Ther..

[11] Depypere M, Morgenstern M, Kuehl R (2020). Pathogenesis and management of fracture-related infection. Clin Microbiol Infect..

[12] Sousa A, Carvalho A, Pereira C (2018). Economic impact of prosthetic joint infection-an evaluation within the portuguese national health system. J Bone Jt Infect.

[13] Levy JF, Castillo RC, Tischler E, Huang Y, O’Hara NN (2020). The cost of postoperative infection following orthopaedic fracture surgery. Tech Orthopaed.

[14] Metsemakers WJ, Smeets B, Nijs S, Hoekstra H (2017). Infection after fracture fixation of the tibia: analysis of healthcare utilization and related costs. Injury.

[15] Ferreira N, Reddy K, Venter RG, Centner CM, Laubscher M (2021). Antibiogram profiles and efficacy of antibiotic regimens of bacterial isolates from chronic osteomyelitis of the appendicular skeleton: a developing-world perspective. South African Med J.

[16] Hellebrekers P, Verhofstad MHJ, Leenen LPH, Varol H, van Lieshout EMM, Hietbrink F (2020). The effect of early broad-spectrum versus delayed narrow-spectrum antibiotic therapy on the primary cure rate of acute infection after osteosynthesis. European J Trauma Emerg Surg.

[17] Mathews CJ, Weston VC, Jones A, Field M, Coakley G (2010). Bacterial septic arthritis in adults. Lancet.

[18] Harris PA, Taylor R, Minor BL (2019). The REDCap consortium: Building an international community of software platform partners. J Biomed Inform.

[19] Harris PA, Taylor R, Thielke R, Payne J, Gonzalez N, Conde JG (2009). Research electronic data capture (REDCap)—A metadata-driven methodology and workflow process for providing translational research informatics support. J Biomed Inform.

[20] Parvizi J, Tan TL, Goswami K (2018). The 2018 definition of periprosthetic hip and knee infection: an evidence-based and validated criteria. J Arthroplast.

[21] Hellebrekers P, Rentenaar RJ, McNally MA (2019). Getting it right first time: the importance of a structured tissue sampling protocol for diagnosing fracture-related infections. Injury.

[22] Rupp M, Baertl S, Walter N, Hitzenbichler F, Ehrenschwender M, Alt V (2021). Is there a difference in microbiological epidemiology and effective empiric antimicrobial therapy comparing fracture-related infection and periprosthetic joint infection? A retrospective comparative study. Antibiotics.

[23] Mthethwa PG, Marais LC (2017). The microbiology of chronic osteomyelitis in a developing world setting. SA Orthopaed J.

[24] Peel TN, Cheng AC, Buising KL, Choong PFM (2012). Microbiological aetiology, epidemiology, and clinical profile of prosthetic joint infections: are current antibiotic prophylaxis guidelines effective?. Antimicrob Agents Chemother.

[25] Lochan H, Moodley C, Rip D (2016). Emergence of vancomycin-resistant *Enterococcus* at a tertiary paediatric hospital in South Africa. South African Med J.

